# A Novel Role for the NLRC4 Inflammasome in Mucosal Defenses against the Fungal Pathogen *Candida albicans*

**DOI:** 10.1371/journal.ppat.1002379

**Published:** 2011-12-08

**Authors:** Jeffrey Tomalka, Sandhya Ganesan, Elaheh Azodi, Krupen Patel, Parth Majmudar, Brian A. Hall, Katherine A. Fitzgerald, Amy G. Hise

**Affiliations:** 1 Center for Global Health and Diseases, Case Western Reserve University, School of Medicine, Cleveland, Ohio, United States of America; 2 Department of Pathology, Case Western Reserve University, School of Medicine, Cleveland, Ohio, United States of America; 3 Department of Medicine, University of Massachusetts Medical School, Worcester, Massachusetts, United States of America; David Geffen School of Medicine at University of California Los Angeles, United States of America

## Abstract

*Candida* sp. are opportunistic fungal pathogens that colonize the skin and oral cavity and, when overgrown under permissive conditions, cause inflammation and disease. Previously, we identified a central role for the NLRP3 inflammasome in regulating IL-1β production and resistance to dissemination from oral infection with *Candida albicans*. Here we show that mucosal expression of NLRP3 and NLRC4 is induced by *Candida* infection, and up-regulation of these molecules is impaired in NLRP3 and NLRC4 deficient mice. Additionally, we reveal a role for the NLRC4 inflammasome in anti-fungal defenses. NLRC4 is important for control of mucosal *Candida* infection and impacts inflammatory cell recruitment to infected tissues, as well as protects against systemic dissemination of infection. Deficiency in either NLRC4 or NLRP3 results in severely attenuated pro-inflammatory and antimicrobial peptide responses in the oral cavity. Using bone marrow chimeric mouse models, we show that, in contrast to NLRP3 which limits the severity of infection when present in either the hematopoietic or stromal compartments, NLRC4 plays an important role in limiting mucosal candidiasis when functioning at the level of the mucosal stroma. Collectively, these studies reveal the tissue specific roles of the NLRP3 and NLRC4 inflammasome in innate immune responses against mucosal *Candida* infection.

## Author Summary

In this manuscript we describe a new role for a group of molecules termed the “inflammasome” that process key immune response proteins including interleukin-1-β. In previous work, we and others have shown that the NLRP3 inflammasome is important in protecting from severe fungal infections. We now show that, in addition to the NLRP3 inflammasome, a different inflammasome containing NLRC4 is also important in protecting against infection with *Candida albicans*, and appears to be functioning in the mucosal lining of the mouth and intestines, rather than in immune cells. Our research explains a new mechanism of mucosal immunity to fungal infections and has broad implications for developing new treatments against fungal infections, which are a serious cause of illness and death, particularly in immunocompromised persons. Additionally, this research may also lead to new ways to identify those individuals who are at the highest risk for serious fungal infections.

## Introduction

*Candida sp.* are dimorphic fungi that commonly colonize the oral cavity of adult humans, with overgrowth prevented by competing commensal bacteria as well as local host immune responses. Perturbations of the normal oral flora through antibiotic treatment, for example, or immunocompromised states can lead to mucosal *Candida* overgrowth resulting in the development of oropharyngeal candidiasis (OPC, also known as thrush). *Candida albicans* has now been identified as the leading cause of fatal fungal infections, with mortality rates as high as 50%, and ranks 4th among all pathogens isolated from bloodstream and nosocomial infections [1]–[3]. Host recognition of *Candida* requires engagement of surface receptors on innate immune cells, including TLR2 and Dectin-1 [4]–[7]. A major consequence of receptor activation is the induction of pro-inflammatory gene expression including interleukin 1 beta (IL-1β), a zymogen which requires proteolytic processing by caspase-1 to become biologically active [8]–[11]. Activation of caspase-1 requires signaling through recently described protein complexes termed inflammasomes, consisting of either NOD-like receptor (NLR) molecules or the PYHIN protein, Absent in melanoma-2 (AIM2) [12]–[16]. NLRs are characterized by the presence of a Leucine Rich Repeat domain, a central NACHT domain involved in oligomerization and protein-protein interactions, and a CARD or PYRIN domain [17]. Conformational changes in NLR proteins, resulting from the introduction of activating stimuli, cause oligomerization of NLR proteins together with ASC adapters, permitting autocatalytic cleavage of pro-caspase-1 to an active state capable of cleaving pro-IL-1β. Although intracellular danger signals and crystalline compounds such as uric acid crystals, cholesterol crystals, amyloid and asbestos have been shown to activate the NLRP3 inflammasome [18]–[22], the precise mechanism(s) underlying inflammasome activation are not defined. Currently, several theories have been proposed for the molecular mechanisms underlying activation of the NLRP3 inflammasome including mitochondrial ROS production [23], phagosomal or endosomal rupture and cell membrane disturbances [24]–[27]. The NLRP3 inflammasome has been linked to IL-1β responses to pathogen-derived molecules including bacterial muramyl dipeptide [28] and toxins [20], [28], as well as in response to a range of bacterial, viral and fungal pathogens, including *Candida albicans* [6], [29]. Another NLR molecule, NLRC4, also forms an inflammasome capable of activating caspase-1 and IL-1β cleavage. During some bacterial infections, such as with *Shigella*, *Salmonella*, *Pseudomonas* or *Legionella*, NLRC4 detects inadvertently translocated flagellin or PrgJ rod protein, a component of the type III secretion system [30]–[35]. Although limited *in vitro* studies using NLRC4 deficient macrophages or dendritic cells challenged with *Candida albicans* revealed no defects in caspase-1-dependent IL-1β responses [29], [36], [37], the role of NLRC4 in live fungal infection models has not been thoroughly defined.

In this study, we sought to examine the role of other inflammasome in anti-fungal defenses *in vivo*. We show that infection with *Candida albicans* leads to up-regulation of NLRP3 and NLRC4 expression in the oral mucosa and this induction is impaired in both NLRP3 and NLRC4 deficient mice. Additionally, we reveal a role for the NLRC4 inflammasome in regulating resistance to mucosal infection with *Candida* as well as preventing systemic dissemination. We show that inflammasome driven IL-1β responses via both the NLRC4 and NLRP3 inflammasome are essential for epithelial antimicrobial peptide production, and other inflammatory responses including IL-18 and IL-17 in response to *Candida* infection. Inflammatory cell recruitment to *Candida* infected oral mucosa is significantly impaired in NLRC4 deficient mice compared to wild-type mice. Using bone marrow chimera mice, we reveal that the activity of NLRC4 is mediated at the level of the mucosal stroma, in contrast to that observed with NLRP3 which is active in both hematopoietic and stromal compartments. Collectively our studies show that, in addition to the NLRP3 inflammasome, there is a tissue specific role for the NLRC4 inflammasome in host sensing and immune defense to non-bacterial pathogens such as *Candida albicans*.

## Results

### Regulation of inflammasome expression in oral mucosal tissues following infection with *Candida albicans*

**Figure 1:**
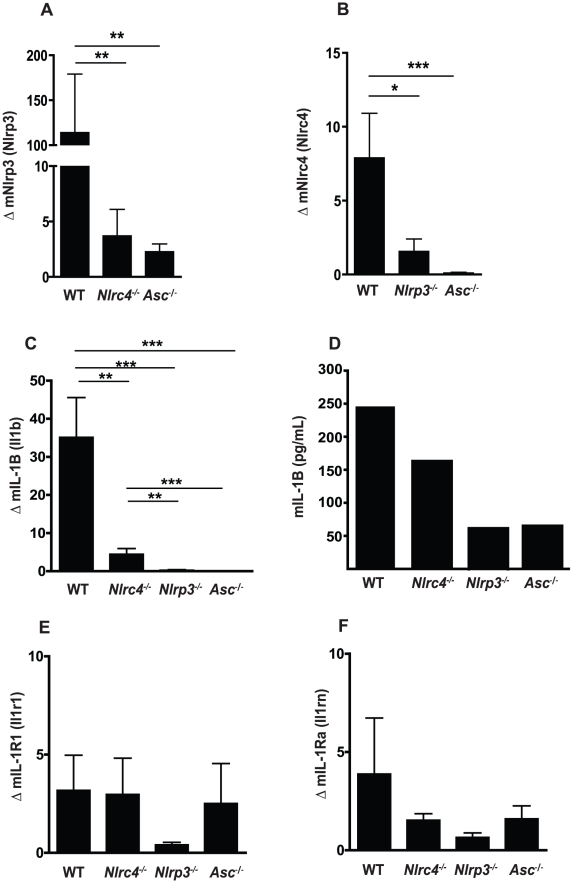
Regulation of *Nlrp3* and *Nlrc4* expression in mucosal candidiasis. Induction of inflammatory genes in buccal mucosal tissues from WT, *Nlrc4 ^−^* ^/*−*^, *Nlrp3 ^−^* ^/*−*^, and *Asc ^−^* ^/*−*^ mice after 72 h of infection with *Candida albicans*. (A) NLRP3, (B) NLRC4, (C) mIL-1β, (E) mIL-1R1, and (F) mIL-1Ra. All genes normalized to GAPDH. (D) Circulating IL-1β levels were determined by ELISA on serum samples pooled from N≥3 animals. (*** *P*≤0.001; ** *P*≤0.01; * *P*≤0.05)

During *Candida* infection, the oral mucosa acts as a physical barrier to infection as well as the initial tissue to respond to fungal growth and invasion. To assess the impact of *Candida* treatment on the oral mucosa, we monitored gene expression levels in oral mucosa by quantitative real-time PCR. We first examined the level of NLR expression in buccal tissues of *Candida* infected mice and observed a strong induction of NLRP3 in wild-type mice following oral challenge with *Candida albicans*. Induction of NLRP3 was significantly reduced in both *Nlrc4^−^* ^/*−*^ and *Asc^−^* ^/*−*^ mice (Figure 1A). Similarly, NLRC4 was induced in WT mice and negligible in *Nlrp3^−^* ^/*−*^ and *Asc^−^* ^/*−*^ mice (Figure 1B). As expected, ASC was not induced in any of the strains after *Candida* infection. These data indicate that genetic knockdown of a single NLR may have profound effects on the expression profile of other NLR proteins and is, to our knowledge, the first evidence of cross-regulation of NLRP3 and NLRC4.

### *Candida* induced IL-1β in the oral mucosa is dependent on both NLRP3 and NLRC4

We next assessed the impact of the NLRP3 and NLRC4 inflammasomes on expression levels of members of the IL-1 family. There was a significant difference in the induction of IL-1β between the WT and *Nlrc4^−/−^, Nlrp3^−^* ^/*−*^, and *Asc^−^* ^/*−*^ mice (Figure 1C). This defect in IL-1β production was confirmed in the serum of infected mice at 3 days of infection (Figure 1D). Levels of IL-1R1 expression were similar between WT and *Nlrc4^−/−^* or *Asc^−/−^* mice with reduced induction observed in *Nlrp3^−/−^* mice, although this was not significant (Figure 1E). Induction of IL-1R antagonist (IL-1Rn) was not significantly different between any of the inflammasome knockout mice and WT mice (Figure 1F). Overall, the induction of IL-1R1 and IL-1Rn was minimal compared to IL-1β in all the infected mice.

### NLRC4 protects against mucosal fungal infection and early dissemination of infection in a murine model of oropharyngeal candidiasis

**Figure 2:**
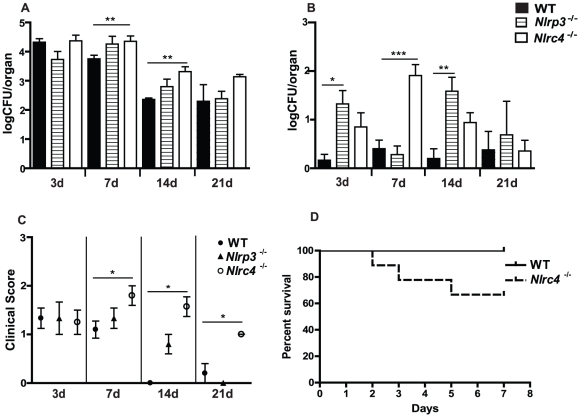
NLRC4 inflammasome protects against mucosal overgrowth and prevents systemic dissemination of infection with *Candida albicans*. (A) Quantitative fungal burden of tongues, and (B) kidneys of *Nlrp3^−/−^, Nlrc4 ^−^* ^/*−*^, and WT mice after oral infection with *C. albicans*; (C) Mean clinical severity score of *Nlrp3^−/−^, Nlrc4 ^−^* ^/*−*^, and WT mice after 3, 7, 14 or 21d of infection. (D) Kaplan-Meier survival curve of WT and *Nlrc4^−/−^* mice infected with virulent *C. albicans.* (*** *P*≤0.001; ** *P*≤0.01; * *P*≤0.05)

Our previous studies demonstrated that NLRP3 signaling is critical for the prevention of fungal growth as well as dissemination in a murine model of oropharyngeal candidiasis [6]. A role for the NLRC4 inflammasome in response to oral fungal challenge has yet to be characterized. In order to ascertain the impact of loss of NLRC4 function on disease progression, we infected wild-type (WT) and *Nlrc4^−^* ^/*−*^ mice with *Candida albicans* as previously described [6]. Oral fungal burdens were elevated in *Nlrc4^−^* ^/*−*^ mice compared to WT mice by day 7, and persistently higher fungal burdens were observed to day 21 (Figure 2A). In our model of persistent, low virulence oral candidiasis, WT mice rarely show blood borne dissemination of infection, as measured by quantitative fungal burdens in the kidneys (Figure 2B). In contrast, *Nlrc4^−^* ^/*−*^ mice show a significantly increased susceptibility to dissemination of infection, peaking at day 7 but returning to WT levels by day 21. In agreement with these findings, *Nlrc4^−^* ^/*−*^ mice also had elevated gross clinical scores, a qualitative measure of oral infection severity, at all time points (Figure 2C). Survival in the *Nlrc4^−^* ^/*−*^ mice was reduced when compared to WT mice when infected with a virulent strain of *Candida albicans* (Figure 2D). Elevated quantitative fungal colonization was observed in tissues of the gastrointestinal tract including esophagus, stomach, and small intestine in *Nlrc4^−^* ^/*−*^ mice compared to WT (Figure S1).

These data contrast with studies in *Nlrp3^−^* ^/*−*^ mice, in which it was determined that oral fungal colonization was similar at day 3, becoming slightly elevated at day 7 and 14, and returning to WT levels by day 21 (Figure 2A). These mice exhibited elevated levels of systemic dissemination throughout the 21 day timecourse (Figure 2B). By day 21, the gross clinical score of both *Nlrp3^−^* ^/*−*^ and WT mice were between 0 and 1, indicating minimal signs of infection, which contrasts the sustained elevated clinical score seen in *Nlrc4^−^* ^/*−*^ mice (Figure 2C). Taken together, our studies imply that NLRC4 and NLRP3 are differentially functioning in the innate response to *Candida* infection, with NLRC4 playing a more prominent role in the clearance of oral infection.

### NLRC4 is required for neutrophil recruitment following *Candida* challenge

**Figure 3:**
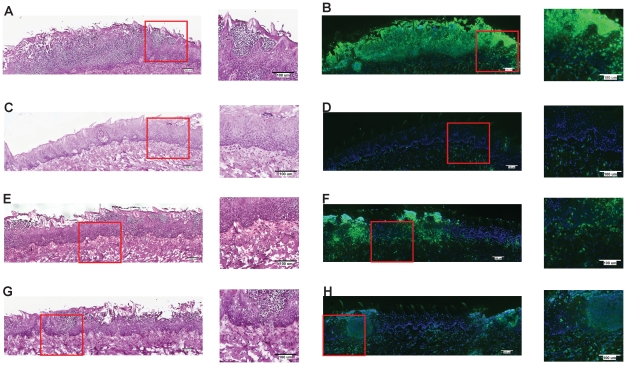
NLRC4 inflammasome mediates neutrophil influx response to mucosal *Candida* infection. PASH stains of GDH2346 infected tongues from (A) WT, (C) *Nlrc4^−/−^,* (E) *Nlrp3^−/−^,* and (G) *Asc^−/−^.* Tongue sections were stained with the neutrophil-specific NIMP antibody and DAPI for (B) WT, (D) *Nlrc4^−/−^,* (F) *Nlrp3^−/−^,* and (H) *Asc^−/−^.* Magnified 40x images of sections are included.

**Figure 4:**
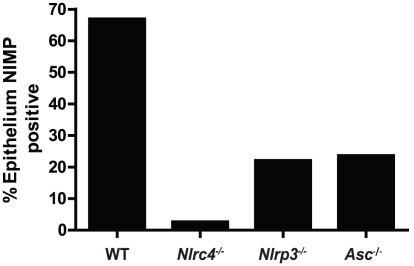
Quantified neutrophil responses in *Candida* infected epithelium. Neutrophil influx into dorsal epithelium of WT, *Nlrc4^−/−^, Nlrp3^−/−^, and Asc^−/−^* mice was digitally quantified using MetaMorph software. Shown are percent ratios of fluorescent pixels (representing neutrophil staining) over total pixels within a region containing the dorsal epithelium and submucosa of infected tongues.

One of the earliest inflammatory cells that migrate to the site of microbial infection are neutrophils, and this chemotaxis is necessary for proper inflammatory responses and anti-microbial defenses. Given the known capacity for IL-1β to mediate leukocyte infiltration into infected tissues, we used histology to examine the impact of inflammasome deficiency on cellular infiltration to the mucosa of the tongue. By day 2, a robust cellular infiltration was observed in the dorsal epithelium of a WT tongue, particularly in areas showing the presence of fungal hyphae and epithelial erosion (Figure 3A). These cells morphologically appear to have multi-lobulated nuclei, consistent with neutrophils. In contrast, minimal cellular infiltration was observed in a tongue from *Nlrc4^−/−^* mouse, despite the presence of erosive lesions and fungal hyphae (Figure 3C). *Nlrp3^−/−^ and Asc^−/−^* tongues exhibited cellular infiltration, although not to the extent of WT; and these areas of concentrated cellular infiltrates also correlated with the presence of fungal hyphae and tissue erosion (Figure 3E, G). Neutrophils have been implicated in the control of a range of microbial infections, including *Candida* [38]–[40]. Given the presence of significant cellular infiltration at 2d post infection, we sought to specifically characterize the extent of neutrophil infiltration in these tissues. Using a monoclonal antibody shown to specifically stain neutrophils, we observed significant neutrophil staining in the outer epithelium of the WT tongue. This immunofluorescent staining localized to the regions of increased cellularity observed in the epithelium with PAS/H staining (Figure 3B). Neutrophils were also observed throughout the sub-mucosal tissue. In agreement with our finding with PAS/H staining, neutrophil influx into the *Nlrc4^−/−^* was drastically reduced (Figure 3D). As expected, a significant influx of neutrophils was observed in both *Nlrp3^−/−^* and *Asc^−/−^* tongues (Figure 3F, H). Intriguingly, it was observed that not only was there a reduction in neutrophil infiltration in the *Nlrc4^−/−^* tongue but the neutrophils present failed to infiltrate the epithelium where the presence of hyphae was detected. This is evidenced by a ∼25 fold reduction in the percent of dorsal epithelium that stains positive for neutrophils in *Nlrc4^−/−^* mice when compared to WT (Figure 4). *Nlrp3^−/−^ and Asc^−/−^* mice showed a ∼3 fold decrease in positive staining (Figure 4). These findings indicate that the activation of *Nlrc4^−/−^* is required for neutrophil recruitment into infected tissues and proper trafficking to the site of active fungal infection.

### Mucosal IL-17 responses to *Candida* are dependent on both NLRP3 and NLRC4

**Figure 5:**
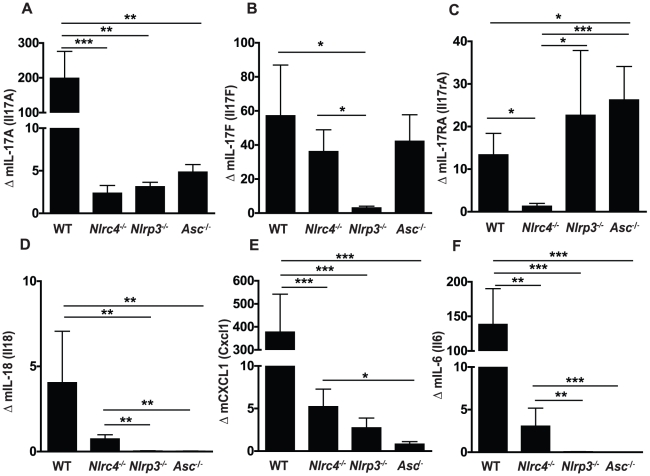
Inflammasome dependence of inflammatory cytokine responses in the oral mucosa following *Candida* infection. Induction of inflammatory genes in buccal mucosal tissues from WT, *Nlrc4 ^−^* ^/*−*^, *Nlrp3 ^−^* ^/*−*^, and *Asc ^−^* ^/*−*^ mice after 72 h of infection with *Candida albicans*. (A) mIL-17A, (B) mIL-17F, (C) mIL-17RA, (D) mIL-18, (E) mKC/CXCL1 and (F) mIL-6. All genes normalized to GAPDH. (*** *P*≤0.001; ** *P*≤0.01; * *P*≤0.05).

Recent reports have implicated the IL-17 family as a critical mediator of protective host responses to a range of extracellular pathogens, including *Candida* [41]–[45]. To assess the impact of inflammasome activation on IL-17 in our model of oral candidiasis, we measured expression levels of IL-17 family members in oral mucosal tissues after infection. A robust increase in IL-17A and IL-17F gene expression was detected in the oral mucosal tissue of WT animals, which was significantly reduced in *Nlrc4^−/−^, Nlrp3^−/−^,* and *Asc^−/−^* mice (Figure 5A, B). In contrast, the induction of IL-17F was dependent on NLRP3, but not NLRC4 or ASC (Figure 5B). A robust induction of interleukin 17A receptor (IL-17RA) expression was detected in WT, *Nlrp3^−^* ^/*−*^, and *Asc^−^* ^/*−*^ mice while this response was abrogated in *Nlrc4^−^* ^/*−*^ mice (Figure 5C). As many downstream inflammatory responses are dependent on IL-17, the failure to upregulate the IL-17A receptor may have implications for local anti-*Candida* inflammation and chemotaxis of inflammatory cells.

### Inflammasome dependence of other inflammatory cytokine responses in the oral mucosa following *Candida* infection

We next examined expression of other inflammatory cytokines in the oral mucosa of mice infected with *Candida albicans*. We observed significantly lower induction of IL-18, another cytokine requiring inflammasome mediated cleavage, in *Nlrp3^−^* ^/*−*^ and *Asc^−^* ^/*−*^ mice, while *Nlrc4^−^* ^/*−*^ mice showed a slight reduction compared to WT which was not statistically different (Figure 5D). As shown in Figure 5E, murine CXCL1, a homolog of human IL-8, was dramatically induced in WT mice following *Candida* infection and levels were significantly reduced in all strains of inflammasome deficient mice. Induction of the pro-inflammatory cytokine IL-6 was also significantly reduced in *Nlrc4^−^* ^/*−*^, *Nlrp3^−^* ^/*−*^, and *Asc^−/−^* mice compared to WT (Figure 5F).

### Mucosal antimicrobial peptide responses to *Candida albicans* infection

**Figure 6:**
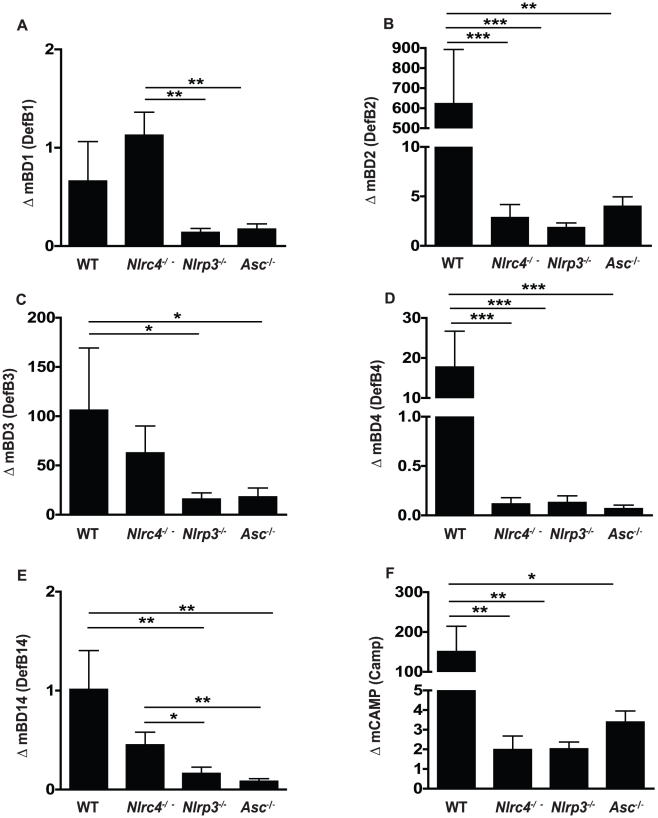
Antimicrobial peptide up-regulation in response to oral infection with *Candida albicans* is impaired in NLRP3, ASC and NLRC4 - deficient mice. Expression of the antimicrobial peptides (A) murine β-defensin 1, (B) murine β-defensin 2, (C) murine β-defensin 3, (D) murine β-defensin 4, (E) murine β-defensin 14, and (F) CAMP in buccal mucosal tissues from WT, *Nlrc4 ^−^* ^/*−*^, *Nlrp3 ^−^* ^/*−*^, and *Asc ^−^* ^/*−*^ mice after 72 h of infection with *Candida albicans*. (*** *P*≤0.001; ** *P*≤0.01; * *P*≤0.05).

In addition to inflammatory cytokines responses following pathogenic encounter, immune cells in the oral mucosa, including epithelial cells, release small antimicrobial peptides designed to disrupt microbial function as well as act as strong chemoattractant signals for the migration of inflammatory cells such as neutrophils and macrophages [46]–[48]. To better define the inflammasome dependence of antimicrobial peptide responses in our murine model of OPC, we quantified several murine beta-defensins as well as cathelicidin responses in the oral mucosa following fungal infection. In contrast to murine beta-defensin 1 (mBD1), which was not induced following infection in any of the mice (Figure 6A), mBD2, -3, -and 4 all showed elevated expression in oral tissues following *Candida* infection in WT mice (Figure 6B, C, D). Reduced or negligible up-regulation in mBD2 and mBD4 gene expression was observed in all of the inflammasome deficient mice (Figure 6B, D). In contrast, mBD3 induction was similar between WT and *Nlrc4^−^* ^/*−*^ mice but significantly reduced in *Nlrp3^−^* ^/*−*^ and *Asc^−^* ^/*−*^ mice (Figure 6C). No appreciable induction of mBD14, a murine homolog of human beta-defensin 3, was observed in any of the mice (Figure 6E). Another antimicrobial peptide, cathelicidin or CAMP, has also been implicated as an activator of the P2X7 receptor and a potential inducer of IL-1β release from cells [49]. Expression levels of CAMP were dramatically elevated in WT buccal tissue following *Candida* infection, and this induction was dependent on NLRC4, NLRP3 and ASC (Figure 6F).

### NLRC4 functioning in the mucosa, compared to NLRP3 in inflammatory cells, is critical for host defense against mucosal colonization

**Figure 7:**
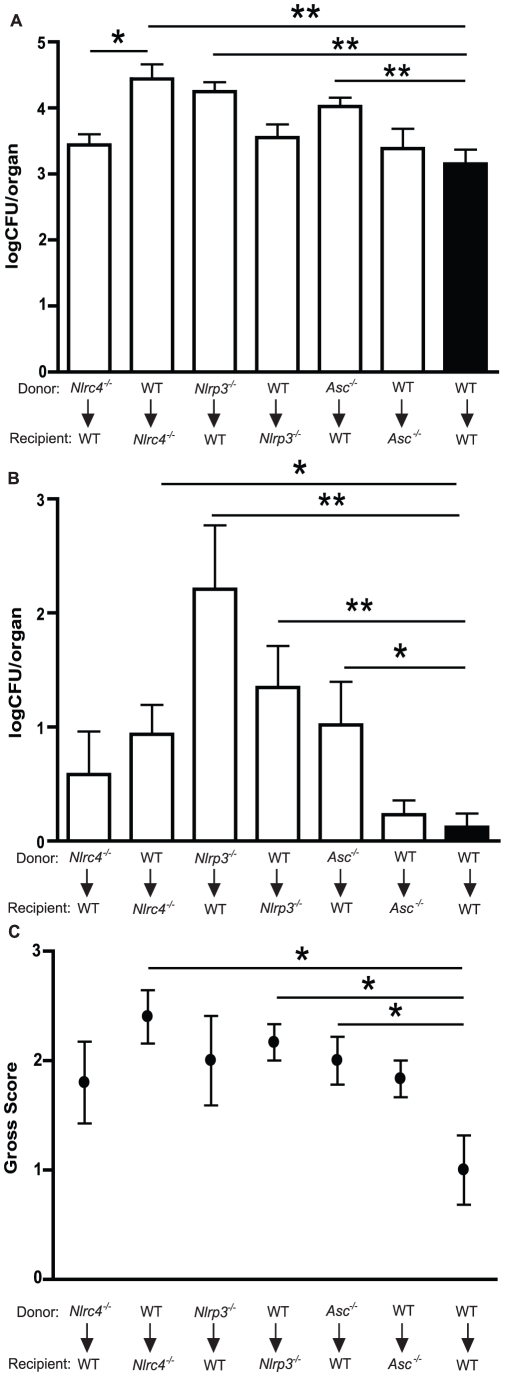
NLRC4 activity in somatic tissues, in contrast to NLRP3 in both hematopoietic and stromal compartments, controls oral infection with *Candida albicans*. Quantitative fungal burden of (A) tongues and (B) kidneys of WT, *Nlrc4 ^−^* ^/*−*^, *Nlrp3 ^−^* ^/*−*^, *Asc ^−^* ^/*−*^ bone marrow chimera mice after oral infection with *C. albicans*; (C) Mean clinical severity score of WT, *Nlrc4 ^−^* ^/*−*^, *Nlrp3 ^−^* ^/*−*^, *Asc ^−^* ^/*−*^ bone marrow chimera mice after 7d of infection. (*** *P*≤0.001; ** *P*≤0.01; * *P*≤0.05).

NLRC4 does not appear to be involved in inflammasome activation in innate immune cells exposed to *Candida in vitro* [29], [36], [37]. Yet in our *in vivo* model on mucosal candidiasis, NLRC4 is required for protection from mucosal colonization, prevention of early dissemination of infection, and neutrophil infiltration. Therefore, we hypothesized that the impact of NLRC4 activation during fungal infection was manifested in mucosal and/or stromal tissues versus hematopoietic cells. The innate immune system is comprised of cells of embryonic origin, including epithelial cells, as well as infiltrating leukocytes derived from the bone marrow. To assess the contribution of different inflammasome molecules in these compartments, we generated bone marrow chimera mice and infected them orally with *C. albicans*. Lethally irradiated recipient mice were reconstituted with bone marrow progenitor cells and allowed to fully reconstitute prior to infection. WT mice that were reconstituted with WT bone marrow exhibited no increase in oral infection or systemic dissemination of infection at 7 days when compared to non-chimeric WT mice, indicating that the chimera procedure does not predispose the mice to higher levels of fungal infection (Figure S2). WT mice reconstituted with *Nlrc4^−^* ^/*−*^ bone- marrow showed no significant difference in oral fungal colonization compared to WT/WT chimera mice (Figure 7A). In contrast, *Nlrc4^−^* ^/*−*^ mice reconstituted with WT bone marrow showed enhanced oral colonization with *C. albicans* (Figure 7A), to a degree that is similar to native *Nlrc4^−^* ^/*−*^ mice (Figure 2A). The *Nlrc4^−^* ^/*−*^ mice reconstituted with WT bone marrow also exhibited increased disease severity when compared to WT/WT chimera mice (Figure 7C). These results demonstrate that intact NLRC4 function in the stromal or epithelial compartment is associated with protection from mucosal infection with *Candida albicans*.

To evaluate the role of the NLRP3 inflammasome in the stromal versus hematopoietic compartments, bone marrow chimeras were generated using *Nlrp3^−^* ^/*−*^ as well as *Asc^−^* ^/*−*^ mice. *Nlrp3^−^* ^/*−*^ mice receiving WT bone marrow showed difference in oral fungal burdens relative to WT/WT chimera controls (Figure 7A). However, WT mice reconstituted with *Nlrp3^−^* ^/*−*^ bone marrow exhibited significantly elevated oral infection when compared to WT/WT chimera mice (Figure 7A), although both sets of NLRP3 chimera mice showed elevated clinical scores compared to WT/WT chimera mice (Figure 7C). A similar pattern was seen with ASC chimeric mice (Figure 7A), where higher oral fungal colonization was observed in the WT mice receiving ASC deficient bone marrow compared to *Asc^−^* ^/*−*^ mice receiving WT cells or WT/WT chimera mice. These observations indicate that the function of the NLRP3/ASC inflammasome complex in infiltrating inflammatory cells is critical for control of oral mucosal infection.

### NLRP3 inflammasome expression in both hematopoietic and stromal cell lineages is required for protection against dissemination of mucosal infection with *C. albicans*

We next assessed the role of NLRC4 and NLRP3 in protection from systemic dissemination of infection. As a marker of dissemination, we quantified the fungal burdens of the kidneys in bone marrow chimera mice infected with *C. albicans* for 7 days. As shown in Figure 7B, neither *Nlrc4^−^* ^/*−*^ donor nor recipient chimera mice showed dissemination to the degree seen in native *Nlrc4^−^* ^/*−*^ mice (Figure 2B), although a trend towards increased dissemination was observed compared to WT/WT chimera mice. In contrast, both *Nlrp3^−^* ^/*−*^ donor and recipient chimera mice showed higher systemic dissemination when compared to WT/WT chimera mice (Figure 7B). WT mice receiving *Asc^−^* ^/*−*^ bone marrow showed enhanced dissemination of infection, whereas *Asc^−^* ^/*−*^ mice receiving WT bone marrow showed similar kidney fungal burdens to WT/WT chimera mice (Figure 7B). These results demonstrate that the NLRP3/ASC inflammasome plays a dominant role in protection against disseminated fungal infection compared to the NLRC4 inflammasome which plays a role in protection of the host from mucosal infection.

## Discussion

Disseminated fungal infections present a significant health risk to both immune-competent and -compromised individuals, making studies into early host immune responses involved in the prevention of dissemination critical for the development of new therapeutic approaches. The release of inflammatory mediators from resident cells at the site of infection is critical for antimicrobial responses including the recruitment of inflammatory cells such as neutrophils and macrophages. IL-1β has been implicated in protective host immune responses to a range of infectious pathogens including viruses, bacteria, and fungi [11]. We and others have previously shown that the NLRP3 inflammasome is important for control of *Candida* infection in both mucosal and disseminated models [6], [29], [36], [37]. Here we present the first experimental evidence implicating the NLRC4 inflammasome in the induction of protective host responses to challenge by a fungal pathogen. Our studies reveal that the NLRC4 inflammasome is important for the control of *Candida albicans* infection *in vivo*, particularly in the oral cavity, with increased oral fungal colonization and disease severity observed in *Nlrc4^−^* ^/*−*^ mice. Lack of NLRC4 also increased susceptibility to disseminated fungal infection, particularly early in infection. Our model of OPC using a clinical isolate recapitulates human oral infections in which mortality is rarely observed. We carried out OPC infection studies using a highly virulent strain of *C. albicans* (ATCC 90234) and observed similar increases in oral colonization and dissemination at 7d to that observed using the oral isolate GDH2346 (Figure S1). However, the virulent strain resulted in significant mortality in *Nlrc4^−^* ^/*−*^ mice when compared to WT (Figure 2E). When we examined cellular infiltrations in infected tongues, we observed a substantial impact in both cellular infiltration, and specifically neutrophil-influx in the absence of NLRC4, compared to a robust neutrophil infiltration into the infected epithelium of WT mice. Interestingly, the neutrophils that were present in the *Nlcr4^−/−^* tongue remained in the sub-mucosa. This defect in neutrophil infiltration into the epithelium of the tongue may explain the extended defect in oral clearance observed in *Nlrc4^−/−^* mice throughout the course of our OPC infection studies. The importance of neutrophils in anti-fungal defenses is well known. Recent studies have shown that the innate inflammatory mileu is critical for effective neutrophil activity against fungal pathogens including IL-6, GM-CSF, and IL-17 responses [38], [41], [42], [50], [51].

In order to better define the molecular basis for protection from mucosal infection, we evaluated a panel of innate inflammatory responses in the oral mucosa from *Candida* infected mice. Previous studies have demonstrated that NLRP3 expression is inducible following infection and implicate this as the rate limiting step in inflammasome activation [52], [53] but little is known about the regulation of NLRC4 expression. IL-1β has been shown to increase the expression level of other pro-inflammatory cytokines following IL-1 receptor engagement [54]-[56], and IL-1 receptor activation may result in the increased expression of inflammasome proteins in the responding cells, priming them for quick activation. We show that *Candida* infection up-regulates NLRP3 and NLRC4 expression in mucosal tissues compared to mock infected mice. Additionally, we demonstrate that this induction is impaired in *Nlrp3^−^* ^/*−*^, *Asc^−^* ^/*−*^ and *Nlrp4^−^* ^/*−*^ mice. As expected, ASC expression was found to be constitutive and not induced by *Candida* infection (data not shown). Our data provides novel insight into transcriptional changes induced by activation of inflammasome complex by an infectious pathogen. The impairment of mucosal NLRP3 induction in NLRC4 deficient mice may enhance the impact of the lack of this receptor on susceptibility to mucosal infection.

As expected, we found that IL-1β was up-regulated in wild-type mice in response to fungal infection. However, these responses were partially abrogated in NLRC4 deficient mice and absent in NLRP3 and ASC deficient mice. Similarly, the IL-1 receptor 1 (IL1R1) and IL-1 receptor antagonist (IL1Rn) were poorly induced by *Candida* infection in WT and inflammasome deficient mice. Consistent with published studies, we did not observe a role for the NLRC4 inflammasome in IL-1β induction or processing in inflammatory cells stimulated *in vitro* with *Candida albicans* [29], [36], [37].

IL-18 is another member of the IL-1 family which requires proteolytic enzyme cleavage for activation [57], [58]. Although caspase-1 mediated cleavage of pro-IL-18 into the bioactive form is the accepted paradigm, alternative mechanisms for cleavage have been proposed including PR3, granzyme B and mast cell chymase [59], [60]. In our studies, *in vivo* IL-18 induction by *Candida* infection showed a similar pattern in mucosal tissues as IL-1β. Next, we investigated the regulation of the cytokine IL-17, which has been shown to play a role in anti-fungal immunity in humans and in animal models [41], [42], [44], [45]. In response to infection with *Candida albican*s, IL-17A, IL-17F and IL-17RA were up-regulated in mucosal tissues. Interesting patterns of induction of the IL-17 family by *Candida* infection were observed; IL-17A was dependent on NLRC4, NLRP3 and ASC whereas IL-17F was only dependent on NLRP3 with comparable induction seen in WT, NLRC4 and ASC deficient mice. The induction of IL-17RA was only dependent on NLRC4. The inflammasome dependence of IL-17 family responses during microbial infection is not well understood, and the regulation of these genes is likely multi-factorial, including dependence on IL-1β as well as other inflammatory mediators. The cytokine IL-6 was induced in wild-type mice in response to *Candida* infection but was significantly reduced in NLRC4 deficient mice and completely abrogated in NLRP3 and ASC deficient mice. The chemokine KC (CXCL1), a murine homolog of human IL-8, was also found to be highly dependent on IL-1β as NLRC4, NLRP3, and ASC deficient mice exhibited significant decreases in gene expression following fungal challenge. Although IL-6 and KC/IL-8 are not known to be major mediators of anti-fungal immunity, they play an important role in the cytokine network of innate cellular communication. IL-6 has been shown to be directly up-regulated in macrophages by *Candida* cell wall components [61] but can also be induced by secondary effect of other inflammatory responses. IL-6 signaling has also been shown to be important in the recruitment of neutrophils in response to *Candida* [38], [62]. IL-6 production is regulated by a complex network of signaling pathways that include NF-κB as well as a newly described pathway mediated by the protein tyrosine phosphatase, Src homology domain 2-containing tyrosine phosphatase-1 (SHP-1) leading to activation of Erk1/2–C/EBPβ [63]. This is particularly relevant to mucosal anti-fungal immunity as SHP-1, also known as PTPN6 and PTP1C, has been shown to negatively regulate the effects of epidermal growth factor [64], an important regulator of epithelial homeostasis, as well as affect tight junction formation in epithelium [65]. We propose, based on our histological analysis, that the defect in the induction of inflammatory mediators observed in mice deficient in the NLRP3/ASC inflammasome is most likely due to defective functioning of inflammatory leukocytes in the absence of these proteins. In contrast, the partial defect in inflammatory responses observed in mucosal tissues from infected NLRC4*^−^* ^/*−*^ mice is likely due to impaired infiltration of immune cells into infected tissue.

Another key innate immune response during infection is the release of antimicrobial peptides designed to limit pathogen growth and survival. Antimicrobial peptides (AMPs) consist of a diverse group of small cationic peptides including the defensins, cationic and amphipathic peptides which have broad antimicrobial and chemotactic properties. Beta-defensins are primarily secreted by epithelial cells and play an important role in the microbial homeostasis of the skin, oral cavity, lung and gut. Human β-defensin (hBD)-1 is primarily expressed in the urinary and respiratory tracts [66], [67] and although constitutively expressed, may be up-regulated by infection or inflammation. A defect in hBD-1 activity in the lung has been associated with cystic fibrosis [68], [69]. Polymorphisms in the defensin-1 gene, *defB1*, have been associated with low oral colonization with *Candida albicans* (Jurevic 2003), protection from HIV [70]–[72], chronic obstructive pulmonary disease [73] and Crohn's disease [74]. The murine homolog of hBD-1, murine β-defensin (mBD)-1, is also expressed by epithelial surfaces, lung and kidney and has salt sensitive antimicrobial activity [75], although its role in antifungal defense is unclear. Both hBD-2 and hBD-3 have known anti-*Candida* [46], [76]–[78] as well as anti-HIV activity [79], [80]. The role of mBD-2, the murine homolog of hBD-2, in oral mucosal health is unclear although its expression in the lung is highly inducible by LPS [81]. The murine ortholog of hBD-3, mBD-14, has inducible expression in the respiratory and intestinal tracts as well as in dendritic cells and shows anti-bacterial and chemotactic activity [82]. To better define the role of AMPs in anti-fungal defense, we examined AMP responses in oral mucosa after infection with *Candida albicans*. We show that mBD-1 appeared to be constitutively expressed in WT mice; however, gene expression was inhibited in NLRP3 and ASC deficient mice. We discovered that mBD-2, -4 and -14 were highly dependent on inflammasome activation as both NLRC4 and NLRP3 as well as ASC deficient mice exhibited dramatically reduced expression levels compared to WT. Interestingly, mBD-3 responses were found to have little dependence on NLRC4 but were dependent on NLRP3 and ASC. Another class of AMPs, the cathelicidins, consisting of human LL-37 and murine CAMP (also known as CRAMP), are known to have anti-*Candida* as well as chemotactic activity [83]–[87]. We observed that CAMP was highly up-regulated in WT mucosa in infected mice, but not in NLRC4, NLRP3 or ASC deficient mice. A recent report found that IL-17A augmented vitamin D3-mediated CAMP production in keratinocytes during psoriasis [88]. In concurrence with these findings, we observed abrogated CAMP expression in NLRC4 and NLRP3 deficient mice, which also lacked IL-17A gene expression, following *Candida* challenge. In addition to its direct antimicrobial effects, CAMP has been identified as a modulator of the P2X7R which has a known role in ATP-induced IL-1β release [49], [89], [90]. From our studies, it can be inferred that initial production of IL-1β may induce IL-17A and CAMP production which can in turn positively regulate further production of IL-1β to create an inflammatory environment which limits fungal infection. This mechanism may serve to explain the strong *in vivo* phenotype observed in our model for NLRC4 and NLRP3 deficient mice, perhaps via a failure to engage this positive feedback loop resulting in an immune state that is prone to persistent infection. In addition to driving IL-1β and IL-18 responses, the NLRC4 inflammasome has been shown to induce a specialized form of programmed cell death, termed pyroptosis or pyronecrosis, characterized by the release of cytoplasmic contents, which include inflammatory mediators such as ATP and arachidonic acid metabolites, to the extracellular matrix. A defect in pyroptosis may partially account for the critical role for NLRC4 activation in our model of candidiasis and provides an opportunity for future research.

Interestingly, our data shows that activation of the NLRC4 inflammasome is important in the stromal compartment, where its role is critical for *in vivo* anti-fungal host defense, but not in the hematopoietic compartment. Using bone marrow chimera mice we differentiated between inflammasome activity in hematopoietic derived cells such as infiltrating macrophages and neutrophils, and embryonic derived mucosal tissues in our model of oropharyngeal candidiasis. This approach demonstrated that NLRP3 and ASC activity in both hematopoietic and stromal compartments are important for protection against oral infection and dissemination. This is in agreement with our previously published findings that the NLRP3 inflammasome was the primary mediator of IL-1β cleavage in murine macrophages stimulated with *Candida in vitro* [6]. In contrast, *in vivo* infection of bone marrow chimera mice showed higher mucosal colonization in NLRC4 deficient recipient mice reconstituted with WT inflammatory cells than WT recipients reconstituted with *Nlrc4* ^-/-^ cells, which had similar levels of oral mucosal infection as WT controls. Overall, these studies utilizing chimera mice in our murine model of mucosal fungal infection implicate a novel, tissue-specific role for the NLRC4 inflammasome.

Many key questions are raised by the findings in this paper. Known microbial activators of the NLRC4 inflammasome include *Salmonella typhimurium* [91], *Shigella flexneri* [92], [93], *Legionella pneumophila* [94] and *Pseudomonas aeruginosa* [34]. Previous reports implicated the activation of NLRC4 by the release of flagellin through the Type-III secretion apparatus and by components of the basal rod proteins of the Type III secretion system itself [93], [95], [96]. Despite these findings, it still remains unclear the mechanism by which NLRC4 senses these activators. Given the homology between the known bacterial activators of NLRC4, it is possible that these proteins may function as a direct receptor recognizing a conserved sequence or structural feature. Current models of NLRP3 activation indicate it does not act as a traditional receptor but rather as a nexus for different pathways invoked following cellular injury and/or infection, which may also be true for the NLRC4 inflammasome. Future studies will seek to elucidate the mechanism of NLRC4 recognition of its activators and also identify the molecule(s) in *Candida* that induce NLRC4 activation. We hypothesize that mucosal NLRC4 activation may occur as an early event in fungal infection, perhaps as a result of cellular damage or direct effect of infection, leading to the induction of innate responses such as anti-microbial peptides and cytokines that recruit inflammatory cells including neutrophils and macrophages that infiltrate the sites of infection. *Candida* induced activation of the NLRP3/ASC inflammasome then provides a critical amplification of the innate response leading to protection of the host from overwhelming mucosal and disseminated candidiasis.

## Materials and Methods

### Ethics statement

The animals described in this study were housed in the AAALAC accredited facilities of the Case Western Reserve University School of Medicine. All animal use protocols have been approved by the Institutional Animal Care and Use Committee of Case Western Reserve University and adhere to national guidelines published in Guide for the Care and Use of Laboratory Animals, 8^th^ Ed., National Academies Press, 2001.

### Fungal preparations

*Candida albicans* strains GDH2346 (NCYC 1467), a clinical strain originally isolated from a patient with denture stomatitis, or ATCC 90234 were utilized for in vitro and in vivo studies. Master plates were maintained on Sabouraud Dextrose (SD) agar. For OPC infection, yeast were grown for 12–16 h in SD broth, pelleted at 3000 rpm for 5 min and washed 2x with sterile 1X PBS. Yeast cells were manually counted using a hemocytometer and diluted to 5×10^7^ cells/mL for live infection.

### Murine model of oral *Candida albicans* infection

Wild-type C57BL/6 mice were purchased from Jackson Laboratories. *Nlrp3^−^* ^/*−*^, *Nlrc4^−^* ^/*−*^ and *Asc^−^* ^/*−*^ mice were generated by Millenium Pharmaceuticals. Animals were housed in filter-covered micro-isolator cages in ventilated racks. Infection and organ harvesting was performed as described previously [6]. Briefly, after pre-treatment with antibiotic containing water, the mice were anesthetized and light scratches made on the dorsum of the tongue following by the introduction of 5×10^6^ *C. albicans* yeast. The scratches are superficial, limited to the outermost stratum corneum, and do not cause trauma or bleeding. After infection of 3 to 21 d, the mice were euthanized, organs harvested and homogenized and fungal burdens assessed by growth on SD agar. For gross clinical score assessment, visual inspection of fungal burdens on the tongues was performed under a dissection microscope. A score of 0 indicates the appearance of a normal tongue, with intact light reflection and no visible *Candida,* a score of 1 denotes isolated patches of fungus, a score of 2 when confluent patches of fungus are observed throughout the oral cavity, and a score of 3 indicates the presence of wide-spread fungal plaques and erosive mucosal lesions.

### RNA extraction

For assessment of inflammatory gene induction, buccal tissue was isolated from infected mice and immediately placed into a RNA stabilization reagent (RNA*later*, Qiagen). After homogenization in lysis buffer for 1.5 min using a bead-beater homogenizer (Retsch), total RNA was isolated using PrepEase RNA Spin Kit (USB/Affymetrix) followed by conversion to cDNA using SuperScript III Reverse Transcriptase (Invitrogen). Whole blood was collected via retro-orbital bleeding into EDTA pre-coated tubes, followed by centrifugation and removal of serum. Serum was stored at −80°C until used.

### Quantitative real-time PCR and enzyme-linked immunosorbent assay

Quantitative real-time PCR was done as described [6]. Specific primer sequences are listed in Table S1. Cytokines were measured in serum by ELISA (R&D). NCBI gene accession numbers are as follows: *Nlrc4*:NM_001033367.3; *Nlrp3*: NM_145827.3; *Asc*: NM_023258.4; *Il1b*: NM_008361.3; *Il1r1*: NM_008362.2; *Il1rn*: NM_031167.5; *Il17a*: NM_010552.3; *Il17f*: NM_145856.2; *Il17ra*: NM_008359.2; *Il18*: NM_008360.1; *Cxcl1*: NM_008176.3; *Il6*: NM_031168.1; *Defb1*: NM_007843.3; *Defb2*:NM_010030.1; *Defb3*: NM_013756.2; *Defb4*: NM_019728.4; *Defb14*: NM_183026.2; *Camp*: NM_009921.2.

### Histology

Intact tongues are removed at necropsy, immediately immersed in Tissue Freezing Medium (EMS) and flash frozen in liquid nitrogen. After cryo-sectioning, 5 µm sections were fixed with10% formalin for 2 min then stained with Periodic Acid Schiff and Hematoxylin (PAS/H). For immunofluorescent staining, the sections were blocked with 5% normal goat serum/PBS, stained with rat monoclonal anti-neutrophil primary antibody (NIMP-R14; specific for Ly-6G and Ly-6C) and Alexa Fluor 488-conjugated goat anti-rat secondary antibody (Invitrogen) and mounted in Vectashield containing DAPI (Vector Laboratories). For quantitative analysis, images were taken using a Leica DMI 6000B inverted microscope, and the number of neutrophils in each section was digitally quantified using the imaging program MetaMorph (Molecular Devices). Briefly, the number of pixels in a region containing the dorsal epithelial portion of the tongue was counted. A threshold value was then assigned which corresponds to a minimum fluorescent value of a neutrophil. The number of pixels at or above this threshold was determined and a percentage of fluorescent pixels determined by dividing by total overall number of pixels.

### Generation of chimera mice

Lethally irradiated mice (exposed to a Cesium-139 γ-radiation source for a total full body dose of 900 rads) received 5×10^6^ bone marrow cells from pooled donor mice via tail vein injection and allowed to recover for 4 weeks.

### Statistical analysis

Data were analyzed using commercial software (GraphPad) and Student's two-sample independent t tests or Mann Whitney U tests were used for comparative statistical analysis of qPCR, ELISA, and quantitative fungal load data. Comparison of survival curves was done using a mean Logrank test. *P* values are presented when statistical significance was observed (significance set at *P*≤0.05 at a confidence interval of 95%).

## Supporting Information

Figure S1 **Quantitative fungal burdens of gastrointestinal tissues from WT and Nlrc4 deficient mice infected orally with** ***Candida albicans*** **.** WT or *Nlrc4^−/−^* mice were infected 7 days. Fungal burdens were determined for (A) esophagus, (B) stomach, (C) duodenum, (D) jejunum, and (E) ileum. (F) Kaplan-Meier survival plot of WT and Nlrc4*^−/−^* mice following GDH2346 infection. (G) Quantitative fungal burden of tongues and kidneys of WT and *Nlrc4^−/−^* mice infected for 7d with virulent *C. albicans* 90234 strain. (*** *P* ≤ 0.001; ** *P* ≤ 0.01; * *P* ≤ 0.05).
(EPS)

Figure S2 **WT-WT chimera mice exhibit similar infection levels to WT non-irradiated WT controls.** Fungal burdens were determined (A) tongue and (B) kidneys. (C) Gross Clinical score was used as a measure of disease severity.
(EPS)

Table S1 **Primers used for measurement of gene up-regulation by real-time PCR.** List of forward and reverse gene specific primers, and PCR product size, used in quantitative real-time PCR.
(TIFF)
